# Exploring cultural influences in perinatal and early childhood nutrition

**DOI:** 10.15446/rsap.V26n3.115569

**Published:** 2024-05-01

**Authors:** Shanti Raman, Sharanya Napier-Raman, María Camila Pinzón-Segura

**Affiliations:** 1 SR: MD. MBBS. FRACP. MAE. Ph. D. Public Health. South Western Sydney Local Health District. University of New South Wales. Sydney, Australia. shanti.raman@health.nsw.gov.au University of New South Wales University of New South Wales Sydney Australia shanti.raman@health.nsw.gov.au; 2 SN: B. A. International and Global Studies. Faculty of Medicine and Health. University of Sydney. Sydney, Australia. snap3609@uni.sydney.edu.au University of Sydney Faculty of Medicine and Health University of Sydney Sydney Australia snap3609@uni.sydney.edu.au; 3 MP: MD. Paediatr. Ph. D. Public Health. Universidad Nacional de Colombia. Bogotá, Colombia. mcpinzons@unal.edu.co Universidad Nacional de Colombia Universidad Nacional de Colombia Bogotá Colombia mcpinzons@unal.edu.co

**Keywords:** Sociological factors, nutritional status, child health, maternal health, perinatal care *(source: MeSH, NLM)*, Determinantes sociales de la salud, estado nutricional, salud infantil, salud materna, atención perinatal *(fuente: DeCS, BIREME)*

## Abstract

**Objective:**

This review analyzes socio-cultural factors impacting maternal and infant nutrition in low-resource settings, covering the perinatal period including pregnancy, childbirth, and early infancy.

**Methodology:**

It examines qualitative studies from 1990 to 2021, identified through databases such as Medline, Embase, and Scopus, using broad search terms including 'traditional beliefs,' 'practices' and 'perinatal'.

**Results:**

The synthesis highlights strong cultural support for breastfeeding across diverse cultures, although traditional taboos and beliefs often undermine exclusive breastfeeding. A deep cultural appreciation for the therapeutic benefits of foods is observed, with prevalent, albeit varied, notions of 'good' and 'bad' foods influencing dietary choices during the perinatal period. Intergenerational support plays a crucial role, though it often conflicts with biomedical advice, particularly in migrant populations. Crosscutting themes include the enduring role of women as 'good mother' in perinatal care, the impact of poverty on nutritional choices, and the evolving nature of cultural practices, the direction of which is not always predictable.

**Conclusions:**

Cultural beliefs profoundly shape perinatal and infant nutrition. It advocates the need for public health strategies that are culturally sensitive and tailored to specific community needs to optimize health outcomes for mothers and infants. Future interventions should integrate cultural understanding into public health practices, promoting beneficial traditions while modifying detrimental ones.

## RESUMEN

**Objetivo:**

La revisión analiza los factores socioculturales que impactan la nutrición materna e infantil en entornos de bajos recursos, abarcando el periodo perinatal que incluye el embarazo, el parto y la primera infancia.

**Metodología:**

Examina estudios cualitativos desde 1990 hasta 2021, identificados a través de bases de datos como Medline, Embase y Scopus, centrándose en temas como 'creencias tradicionales', 'prácticas' y "perinatal".

**Resultados:**

La síntesis resalta un fuerte apoyo cultural para la lactancia materna en diversas culturas, aunque los tabúes y las creencias tradicionales a menudo socavan la lactancia materna exclusiva. Se observa una profunda apreciación cultural por los beneficios terapéuticos de los alimentos, con nociones prevalentes, aunque variadas, de alimentos 'buenos' y 'malos' que influyen en las decisiones dietéticas durante el periodo perinatal. El apoyo intergeneracional desempeña un papel crucial, aunque a menudo entra en conflicto con el asesoramiento biomédico, particularmente en poblaciones migrantes. Los temas transversales incluyen el papel perdurable de las mujeres como "buenas madres" en atención perinatal, el impacto de la pobreza en las elecciones nutricionales y la naturaleza evolutiva de las prácticas culturales, cuya dirección no siempre es predecible.

**Conclusiones:**

Las creencias culturales moldean profundamente la nutrición perinatal e infantil. Se aboga por la necesidad de estrategias de salud pública que sean culturalmente sensibles y adaptadas a las necesidades específicas de la comunidad, para optimizar los resultados de salud para madres e infantes. Las futuras intervenciones deben integrar la comprensión cultural en las prácticas de salud pública, promoviendo tradiciones beneficiosas mientras se modifican las perjudiciales.

The perinatal period is crucial for health interventions and significantly contributes to the disease burden in low-resource settings 1. The first 1,000 days from conception to two years of age are critical for infant growth faltering 2. Factors such as maternal education, health, nutrition, and social determinants like poverty, illiteracy, and the status of women, are fundamental to child outcomes. However, there is a shortage of community-based intervention studies in these settings.

Maternal and child malnutrition remains problematic, causing nearly three million child deaths annually, and maternal and childhood overweight and obesity also contribute to the chronic disease burden 3. Accessing appropriate nutrition for women and children is challenging. The perinatal period offers a key opportunity to address undernutrition and its adverse effects, but cultural, social, and psychosocial factors play a significant role in the perinatal experience, including parenting practices that can have long-term benefits or harms on children's development.

Following Nichter's landmark study on childhood malnutrition in India 4, research has demonstrated how maternal dietary and infant feeding practices are shaped by local cultural norms and constraints. Our study explores qualitative research in low-resource settings about the perinatal period, focusing on cultural beliefs, values, and practices, aiming to identify common themes and gaps in the research. This review focuses on qualitative studies from low- and middle-income countries (LMICS) but includes migrant populations in high-income countries to reflect current situations for women and children.

## METHODS

We expanded our systematic review, previously described in Raman et al. 5, with a revised search strategy for this narrative review.

### Selection Criteria

Initially, we sought studies with primary qualitative or mixed-method data (interviews, focus groups, ethnography) on cultural practices and beliefs affecting the perinatal period in LMICS, excluding articles that used structured questionnaires, lacked information on cultural practices, or focused on high-risk conditions like HIV or diabetes. For the updated search, we included studies beyond LMICS and excluded non-English articles to avoid translation biases.

### Literature Search

*Phase 1:* We systematically searched databases from 1990 to 2014, including Medline, Embase, Cochrane Library, and others, complemented by hand searches. Keywords included "perinatal", "pregnancy", "childbirth", "cultural beliefs", and "low resource setting". *Phase 2:* An updated search through Medline, Embase, and Scopus incorporated studies post-2014, focusing on maternal and infant nutrition and removing certain geographic terms to include migrant and refugee communities in developed nations.

### Quality of Reporting

We applied the CASP Qualitative Research Checklist 6 to assess the explicitness and comprehensiveness of reporting in both review phases.

### Data Synthesis and Analysis

We synthesized the data using thematic synthesis methods 7, involving line-by-line coding, organizing codes into themes, and abstracting findings to produce new interpretations.

## RESULTS

### Literature search

**Figure 1:**
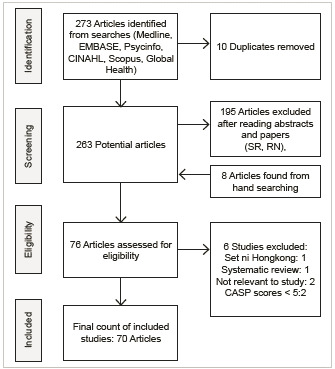
Phase 1: Flow diagram of search and study inclusion process

**Figure 2:**
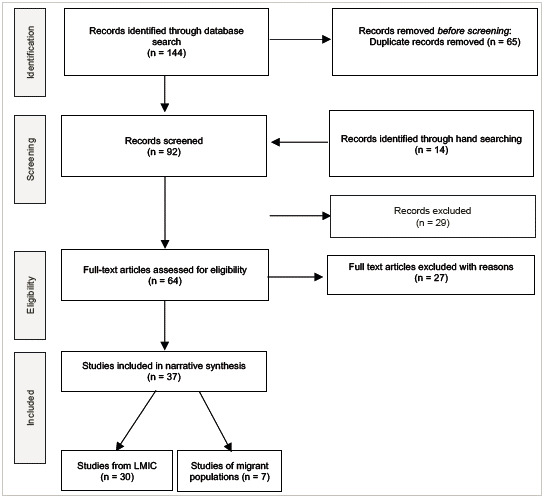
Phase 2: Flow diagram of search and study inclusion process

Initial search found 273 papers, 76 eligible studies; 70 were included (Figure 1). Phase 2 search found 144 papers, 64 eligible, and 37 included; 30 were from LMIC, 7 from developed countries' migrant or refugee populations (Figure 2). Table 1 shows LMIC studies (100 included), and Table 2 shows high-income settings' migrant studies by region, author, year published, and quality scores. The mayority of studies were from Africa and Asia, and published after 2010 (Table 3). Additionally, there was widespread belief in the healing properties of certain foods and medicines used during the perinatal period (Table 4).

LMIC: Low and middle-income countries

LMIC: Low and middle-income countries.

**Table 1:** Included studies by region, author, year and quality scores- Low and middle-income countries

Setting	Author (Year)	CASP Score
Africa		
Ghana, rural	Arzoaquoi (2015)	9
Nigeria, rural	Asowa-Omorodion (1997)	7
Uganda, rural	Ayiasi (2013)	8
South Africa, rural	Chakona (2019)	9
Mozambique, peri urban	Chapman (2003)	9
Ghana, urban	Dako-Gyeke (2013)	9
Ghana, urban	de-Graft Aikins (2014)	6
Ethiopia, rural	Degefie (2014)	8
Burkina Faso, rural	Donmozoun (2014)	6
Sierra Leone, rural & urban	Dorwie (2014)	10
Nigeria, rural & urban	Ejidokun (2000)	10
Ghana, rural & urban	Farnes (2011)	10
Malawi, rural	Flax (2015)	9
Ethiopia, rural	Gebrehiwot (2012)	9
Kenya, rural & urban	Geissler (1999)	7
Tanzania, rural	Gross (2013)	8
Uganda, rural	Ickes (2017)	9
Ghana, periurban and rural	Kalra (2018)	8
Uganda, rural	Kwagala (2013)	9
Uganda, rural	Kyomuhendo (2003)	7
Tanzania, rural	Lennox (2017)	8
Benin, rural, peri-urban	Lokossou (2021)	8
Liberia, rural	Lori (2011)	10
Zambia, rural & urban	Maimbolwa (2003)	10
Zambia, rural and urban	Maliwichi-Nyirenda (2016)	5
Tanzania, urban	Mbekenga (2013)	9
Ghana, rural	Mills (2005)	9
Kenya, rural and urban	Mohamed (2020)	8
Ghana, rural	Moyer (2014)	9
S Africa, rural	Ngomane (2012)	9
Nigeria, rural	Orisaremi (2013)	9
S Africa, rural	Preez (2012)	8
Kenya, rural	Riang'a (2017)	9
Kenya, rural	Riang'a (2017)	9
Ghana, peri urban	Theroux (2013)	7
Swaziland, rural	Thwala (2011)	7
Swaziland, rural	Thwala (2012)	8
Ethiopia, rural	Tsegaye (2021)	10
Cameroon, rural	van der Sijpt (2013)	9
Ethiopia, rural & urban	Warren (2010)	6
Ghana, rural	Wilkinson (2010)	9
DRC, urban	Wood (2020)	9
Ethiopia, rural	Zerfu (2016)	9
Malawi, rural	Zulu (2001)	8
South Asia		
Bangladesh, urban	Ahmed et al. (2010)	9
India, urban	Athavale (2020)	9
Pakistan, rural	Baloch (2020)	7
India, rural	Bandyopadhyay (2w009)	7
Nepal, peri urban	Brunson (2010)	9
India, rural	Chanchani (2019)	9
India, rural	Chakrabarti (2019)	7
Bangladesh, rural	Choudhury (2011)	9
Bangladesh, urban	Choudhury (2012)	8
India, rural	Craig (2018)	9
India, rural	Debnath (2021)	7
Pakistan, rural	Dykes (2012)	7
Pakistan, urban	Fikree (2004)	8
Pakistan, urban	Fikree (2005)	7
India, rural	Iyengar (2008)	8
Nepal, rural	Kaphle (2013)	10
India, rural	Kesterton (2009)	8
Pakistan, rural	Khadduri (2008)	7
Bangladesh, urban	Moran (2009)	9
Bhutan, urban	Pemo (2019)	9
Pakistan, rural	Premji (2014)	10
India, urban	Raman (2014)	10
Bangladesh, urban	Rashid (2007)	8
India, rural	Sharma (2013)	10
Nepal, rural	Sharma (2016)	10
Nepal, rural	Thapa (2000)	7
Bangladesh, rural	Winch (2005)	8
Asia, other		
Tibet, rural	Adams et al. (2005)	10
Laos, rural	Alvesson et al. (2013)	9
Indonesia, peri-urban	Astuti (2021)	10
Laos, rural	de Sa et al. (2013)	8
Turkey, urban	Ergenekon-Ozelci (2006)	7
Philippines, peri urban	Hadwiger (2012)	9
China, urban	Kartchner, Callister (2003)	9
Laos, urban	Lee et al. (2013)	10
Vietnam, urban	Lundberg,Ngoc Thu (2012)	9
Vietnam, urban	Lundberg (2011)	10
Cambodia, rural	Matsuoka et al. (2010)	8
China, rural	Raven et al. (2007)	9
Myanmar, rural & urban	Sein (2013)	10
China, rural	Strand et al. (2009)	9
Laos, rural	Sychareun et al. (2012)	8
Indonesia, rural	Tobing (2019)	8
Cambodia, rural	White (2002)	9
Cambodia, rural	White (2004)	9
Bali, rural	Wulandari (2011)	9
Middle East		
Syria, urban	Abushaikha (2013)	8
Egypt, urban and rural	Kavle (2018)	9
Jordan, urban	Khalaf, Callister (1997)	8
Iran, urban	Nasrabadi (2019)	7
Latin America		
Guatemala, rural	Berry (2006)	8
Colombia, rural	Concha (2021)	9
Argentina, urban	Hess and Maughan (2012)	8
Brazil, rural	Piperata (2008)	10
Guatemala, rural	Radoff et al. (2013)	10
Peru, rural	Nuño Martínez (2021)	9

CASP: Critical Appraisal Skills Programme, scored out of 10.

**Table 2:** Included studies by region, author, year and quality scores- Migrant populations

Setting	Population group	Author (year)	CASP Score
Canada, urban	Chinese	Higginbottom (2018)	8
United States, rural	Hispanic	Hohl (2016)	9
Australia, urban	Vietnamese and Myanmarese refugees	Joseph (2019)	10
United States, urban	Chinese	Lee (2014)	9
United Kingdom, urban	Chinese	Leung (2017)	8
United states, urban	Latinx	MacMillan Uribe (2021)	
Ireland, urban	Chinese	Zhou (2020)	10

CASP: Critical Appraisal Skills Programme, scored out of 10.

**Table 3:** Summary of included studies by region, time period and quality scores

Region (N)	Time Frame (N)	CASP Scores
Africa 44	1990-1999 2	7
	2000-2009 6	7-10
	>2010 36	5-10
South Asia 27	2000-2009 10	7-9
	>2010 17	8-10
Asia (Other) 19	2000-2009 7	7-10
	>2010 12	8-10
Latin America 6	2000-2009 2	8-10
	>2010 4	8-10
Middle East 4	1990-1999 1	8
	>2010 3	7-9

**Table 4:** Examples of ethno-medicine/healing foods used by women during the perinatal period

Author	Region	Food substances	Effect
Adams et al. (2005)	Tibet	Butter ingested by newborn *Chang* warm barley beer ingested by mothers	So, child will have a clear mind and well-developed senses
Ayiasi et al. (2013)	Uganda	*Waragi* local alcohol	Generally therapeutic, keeps infant's skin clear
Farnes et al. (2011)	Ghana	Local herbs ingested by mothers	Prevents *sunsumyare* (spiritual sickness), promotes maternal, fetal health, prevents complications
Hadwiger and Hadwiger (2012)	Philippines	Ginger, carried	Protect unborn baby from *aswang* (evil spirits)
Lundberg and Trieu Thi Ngoc (2011)	Vietnam	Pig's trotter with papaya or red bean and potato, meat and eggs	Enrich blood, help recovery, encourage expulsion of the lochia, stimulate lactation
Maimbolwa et al. (2003)	Zambia	Traditional medicine applied to vagina	Prepare and widen the birth canal in pregnant women
Ngomane and Mulaudzi (2012)	S Africa	*Mbita, Ritlangi, Mpundulo Mbheswana,* roots of *Xirhakarhani,* boiled *Dinda*	Strengthen and preserve pregnancy Induction, management of labour and management of pain
Radoff et al. (2013)	Guatemala	Teas and baths from grasses and trees, cypress, pine, oak, pear, eucalyptus	Stimulate labour, reduce postpartum bleeding
Raven et al. (2007)	China	Ginger and wine Meat and eggs	Enrich blood, help recovery, encourage expulsion of the lochia, stimulate lactation
Sein (2013)	Myanmar	Turmeric, ingested or applied on skin	Prevent muscle pain and to prevent newborn from abdominal pain
Thapa et al. (2000)	Nepal	Mustard oil, turmeric, eggs ingested	Regain energy post-partum, make womb strong, relieve pain
Theroux et al. (2013)	Ghana	Bitter leaf, dandelion, *prekos, maringa, nim* tree, and *kontosi Foufou* pounding	Treat minor illness and maintain/improve pregnancy Prepare for labour
Wulandari and Klinken Whelan (2011)	Bali	Tamarind, turmeric, cinnamon, clove, coconut Herbal medicines	Improve maternal and infant health

#### Thematic Synthesis

The following themes relating to cultural practices and beliefs influencing perinatal and infant nutrition were identified.

#### Breastfeeding: "Everyone here breastfeeds their babies" 8

Breastfeeding is widely supported across cultures, viewed as superior to all other feeds and often described as a "gift from God" 9, including among Hispanic migrants who strongly identify with breastfeeding 10. Despite this cultural endorsement, practices vary. Common practices include preparing and cleaning the breast and infant 8, and using traditional remedies like ash massage in rural Laos 11. In Bhutan, a mother stated, "Not breastfeeding never occurred to me, it's the best and natural" 12.

However, taboos and prohibitions also exist. Islamic teachings recommend breastfeeding for up to two years 13, while other beliefs suggest that pregnant women's milk is harmful 14. Pre-lacteal feeds vary regionally, with practices in South Asia including giving honey, mustard oil, or goat milk 15,16, and in Bangladesh, sugar water and banana 17. In Pakistan, a traditional feed involves honey and butter 18, believed to imbue the baby with certain qualities. In Uganda, the practice differs with age; younger mothers give glucose, whereas older mothers prefer water and salt 19. A concerning belief in parts of Africa is that newborns must consume water daily, leading some mothers to dilute breast milk 20.

In Peru, breastfeeding is crucial for development, though some associate it with causing diarrhea in certain contexts 21. Nigerian beliefs about breastfeeding have evolved with access to modern medications mitigating previous concerns 22. In Africa, traditional tests for milk safety, such as placing an ant in the milk, still occur; if the ant dies, the milk is deemed harmful 23.

Colostrum is often rejected due to beliefs about its quality and effects 14,16. Discrepancies between elder wisdom and medical advice are common, with elders often promoting early food supplements contrary to health recommendations 11. Elders typically influence breastfeeding practices significantly, including the timing of introducing additional foods 24.

#### Healing Foods and Medicines: "Godds own way of helping the baby" 25

Widespread beliefs were found about the healing properties of certain foods and medicine in the perinatal period; especially in more traditional populations in Asia and Africa (Table 4). For example, in Myanmar, nearly all women use turmeric to alleviate muscle and newborn abdominal pain, and licensed traditional medicines are available in hospitals to enhance postpartum recovery and lactation 26. In Cambodia, women drink Khmer medicine to expel residual blood post-delivery 27, while in Zambia, traditional medicines are used to facilitate labor if it is prolonged due to stress or infidelity 28.

Herbalists and traditional healers, including witch doctors and religious leaders, play a significant role in these practices. They are respected for promoting autonomy and are often seen as providers of "God's medicine" 25. In Kenya, Maasai women use local herbs not only for health but to cleanse the body post-dietary indulgence (29), while Ghanaian women prefer herbal medicine over pharmaceuticals, citing strength versus weakness 30. Traditional food and drink are also part of the rituals for newborn care in Tibetan and Balinese cultures 31,32.

Soil-eating, particularly among pregnant women, is another practice noted for its supposed health benefits, affecting the blood's quality and thus, the overall health and fertility, with cultural approval varying significantly across genders and regions 33.

#### "Good food versus bad food: 'there are bad foods and good foods to consume, and I want to consume good foods'"^34^

The study shows a broad spectrum of foods categorized as 'good' or harmful across different cultures. In Asia, including South Asia, traditional beliefs classify foods as "heaty" or cooling, a distinction that persists even post-migration 35. In many parts of Asia, post-partum practices involve consuming 'hot' foods to restore balance after childbirth, which is believed to deplete both yin and yang 36. In China, traditional Chinese Medicine influences the consumption of 'hot' foods like meat and eggs, often enhanced with ginger and wine 37. Similarly, Vietnamese new mothers consume boiled vegetables and pork-based soups 38, while Balinese prefer vegetables to improve the quality of breast milk 32.

In Bengal, post-partum diets include milk, ghee, and fish, supplemented with garlic to aid in uterine recovery or as is described "drying of the womb" 16. The Philippines promotes a diet of boiled vegetables during pregnancy to strengthen both mother and fetus, steering clear of unhealthy fats and sugars 39. In Zambia, nourishing local foods like vegetables with groundnuts and *nshima* (maize flour) are emphasized for maternal health 28.

Dietary restrictions are prevalent during pregnancy and lactation. A Ghanaian woman shared that ignoring food taboos could lead to spiritual consequences affecting the pregnancy 40. In Pakistan, new mothers are advised against eating rice, prawns, and fish to prevent abdominal pain, whereas certain foods are recommended to encourage healthy post-partum bleeding 41. In South Africa, isiXhosa women are often restricted from consuming nutritionally valuable foods like fruits and meats during pregnancy as per traditional advice 42.

The classification of most fruits and vegetables as 'cold' can lead to beliefs about their negative effects, such as causing infant and maternal diarrhea and other discomforts 37. South Asian women also believe 'cold' foods, such as yogurt, cold water during the puerperium can have long-term negative health consequences including "backache, body aches, weakness and fever" 43. In rural Bengal, post-partum prohibited foods include various vegetables and fruits believed to hinder recovery 16. In Nepal, certain 'cold' foods are avoided post-delivery to prevent child diarrhea 44. Cultural taboos in Cambodia restrict many common foods, impacting maternal diet significantly 8. In Zambia, specific dietary advice includes avoiding eggs to prevent a baby being born without hair and avoiding fish to avoid a large anterior fontanelle 27.

Eating excessively or the wrong types of food, leading to a 'big baby', is viewed as dangerous in many cultures, including Ethiopia, where reducing food intake is practiced to facilitate safer childbirth 45. Additionally, Ethiopian pregnant women avoid dairy products to prevent potential harm to the fetus 46. Maasai women avoid sweets, beans, and milk, believing that these foods contribute to fetal overweight 29.

#### Restrictive practices influencing nutrition: "They don't let you eat, you can't eat salt, oil, ...you can't eatyourfill" 47

"Restrictive practices influencing nutrition: 'They don't let you eat, you can't eat salt, oil, .. .you can't eat your fill'" 47. These practices, common in Asia, Africa, and parts of Latin America during the perinatal period, impact food, physical activity, and mobility. A Nepalese health worker reports that new mothers are restricted from salt, green vegetables, and sunlight until the nwaran purification day 48. In rural India, a new mother's diet is limited to dry foods like rice crisps and ghee, consumed only once daily to aid uterine contraction 16. In Nigeria, traditional healers dictate restrictions on pregnant women's mobility and diet, often including nutrient-rich local foods 49. Post-partum confinement in Southeast Asia, known as zuo yuezi in China, involves strict dietary limitations to foods like millet soup and eggs, aiming to help mothers regain strength, although it often leads to physical weakening and mental health issues 47,50. Similarly, Brazilian indigenous women's resguardo involves avoiding certain meats and fruits believed to cause illness, lasting up to 41 days depending on the baby's gender 51. In Myanmar and Cambodia, women avoid cold and strenuous activities to prevent health issues like toas, characterized by symptoms such as diarrhea and abdominal pain 26,27. Post-partum diets in Bangladesh include dry foods and spices believed to cool the stomach and boost milk production 52. In rural Vietnam, only 'hot' or 'warm' foods are recommended to strengthen new mothers, with 'cold' foods being avoided 53.

#### ***"*Social and inter-generational support: 'oh, she [grandmother] can be tiresome ... but my mother's support has been essential to my baby's upbringing'"**^54^

Family and extended female networks play a crucial role in breastfeeding and early childhood nutrition. A Maasai woman highlighted the guidance from elder women with childbirth experience 29. In Indonesia, a young mother valued her mother's ongoing support in teaching breast-feeding and infant feeding techniques 55. Conversely, Nasrabadi found that in urban Iran, spousal support significantly impacted breastfeeding success, with lack of support linked to lower breastfeeding rates 56. Conflicts can arise, particularly when traditional practices clash with biomedical advice, as noted by an Indian mother who felt pressured by her mother-in-law's traditional views 57. Another Indonesian mother experienced distress when her mother-in-law fed her newborn solid food prematurely 55.

#### Cross-cutting themes

Highlighting societal factors critical for understanding maternal-infant nutrition, including among migrant populations in Western countries.

#### Role of woman/mother/wife as strong and good: "as women we just bear that burden, that's all" 47

In traditional societies, a woman's value often hinges on her role in reproduction and endurance during childbirth, as seen in Uganda's Sabiny community 58. For example, Khmer women adhere to restricted diets post-partum despite extreme hunger 8, and a Bhutanese mother describes enduring pain during breastfeeding 12.

#### Poverty and its pervasive effects on perinatal nutrition: "We mostly eat the cheapest available food" 13

Many women in resource-limited settings understand the importance of nutritious food but cannot afford it. A study in urban Ghana found mothers unable to buy essential vegetables 59, while pregnant Kenyans predominantly consumed carbohydrates due to financial constraints 33. Similarly, Egyptian women often relied on readily available foods like beans and potatoes 60. In rural Bengal, only economically better-off women accessed 'special' foods during the perinatal period 16. Financial pressures also affect breastfeeding, highlighted by an Iranian mother forced back to work postpartum due to lack of maternity leave. Migrant women in the West struggle with cultural dietary practices and returning to work soon after childbirth due to financial necessity and lack of traditional support 35,50,61,62.

#### "Change is constant but unpredictable: 'But now we have forgotten our herbs because there are hospitals around'" 23*.*

This theme emerges clearly in evolving breastfeeding and childcare practices, especially among migrants in the West, caught between traditional and biomedical advice 62. Despite awareness of colostrum benefits, South Asian mothers often do not practice exclusive breastfeeding 63.

Cultural practices are dynamic; young mothers sometimes reject the advice of older generations, despite potential repercussions like losing further support from their mothers-in-law 64. Pregnant or lactating mothers blend traditional and Western medicines, exemplified by a Ghanaian mother combining herbs with prescribed drugs 65. In Chattisgarh, India, while women accept immunizations, they reject micronutrient pills fearing they will lead to cesarean sections 66. Conversely, Chinese migrants in Canada adopt non-traditional elements like cow's milk to promote infant growth 35. However, inappropriate biomedical practices, such as unnecessary milk powder prescriptions in Iran, hinder natural breastfeeding 56.

Cultural practices like "doing the month" evolve, with Chinese migrants adjusting traditional dietary restrictions due to time constraints 67. Swazi mothers might use traditional medicines postpartum under familial pressure 68. Latina mothers in the U.S. selectively integrate cultural practices with biomedical advice to benefit their children 69.

Positive changes in breastfeeding practices are evident, although slow. An older Ugandan mother avoided supplements until her milk came 19, and mothers in Kinshasa and Neyshabour follow medical advice strictly regarding breastfeeding, even against community pressure 20, 56.

## DISCUSSION

Our review of qualitative studies from the past three decades shows that cultural influences strongly affect maternal and infant nutrition, often outweighing biomedical advice. Significant cultural support exists for health-promoting practices like breastfeeding, recognizing the healing properties of foods, and the need for rest during the perinatal period. Our findings indicate that while social support is crucial for maintaining these practices, it sometimes comes with challenges, particularly from the older generation, who may resist change. This tension reveals a gendered aspect of cultural transmission, often placing more restrictions on women 70.

Despite strong cultural backing for breastfeeding, global data shows a decline, with early initiation at 52% and exclusive breastfeeding at less than a third for 4-5 months in certain regions. This decline is exacerbated by aggressive marketing of breastmilk substitutes and globalization 71,72. Our review also highlights the persistence of culturally mandated dietary practices, such as the concepts of 'heating' and 'cooling' foods in Asia, which are more influential than biomedically supported nutrition choices 35.

The role of social support, especially from older women like mothers and mothers-in-law, remains significant in influencing infant feeding practices. However, restrictive practices that curtail women's mobility and nutrition, such as resguardo and zuo yuezi, may hinder rather than help postpartum recovery, impacting both physical and mental health 47,73. Interestingly, in some cultures, such as among the Tsimane in Bolivia, modernization has unexpectedly intensified traditional practices like breastfeeding 74. The addition of studies on migrant populations emphasized the impact of poverty on accessing supportive interventions 50,60.

Our review identified a gap in studies from Latin America and the Middle East, possibly due to language barriers in the literature search. The focus of existing research tends to emphasize negative traditional practices rather than positive aspects of culture in perinatal care.

In conclusion, the diversity of cultural practices impacting perinatal nutrition confirms that generalizations are not feasible, and comparisons across groups and regions can be problematic. It is essential to consider cultural dimensions in perinatal and early childhood nutrition. Public health policymakers and clinicians should design interventions that respect cultural practices and promote beneficial behaviors while addressing harmful traditions, emphasizing the importance of culture in shaping health outcomes ♦
